# A Comparative Study of Osteoarthritis Knee Arthroscopy versus Intra-Articular Platelet Rich Plasma Injection: A Randomised Study

**DOI:** 10.5704/MOJ.2207.004

**Published:** 2022-07

**Authors:** N Singh, V Trivedi, V Kumar, NK Mishra, S Ahmad, SJ Ayar, SS Kataria, H Kharbanda

**Affiliations:** 1 Department of Orthopaedics, Era's Lucknow Medical College and Hospital, Lucknow, India; 2 Department of Pathology, Era's Lucknow Medical College and Hospital, Lucknow, India

**Keywords:** osteoarthritis, PRP, arthroscopy, Kellgren-Lawrence, WOMAC

## Abstract

**Introduction:**

Osteoarthritis (OA) is estimated to be the fourth leading cause of disability in the general population. It probably is the most common disease of joints in adults throughout the world. Knee OA accounts for more than 80% of the disease’s total burden and as per an estimate in US population, it affects at least 19% of adults aged 45 years and older. This was a randomised study aimed to evaluate the efficacy of platelet rich plasma (PRP) as a treatment modality for osteoarthritis knee in comparison to arthroscopic management.

**Materials and methods:**

This study was conducted from 2018 to 2020 at a tertiary care teaching hospital, under reference number ELMC&H/RCELL2019/39. A total of 70 patients of osteoarthritis knee with grade 2-3 according to the Kellgren-Lawrence classification were selected using computer generated random number among them 35 patients were subjected to arthroscopy (Group II) and 35 were administered platelet rich plasma injection (Group I) and evaluated at 3, 6 and 9 months of follow-up. Both the groups were assessed and scored with the Western Ontario and McMaster Universities Arthritis Index (WOMAC) and Visual Analog Pain Scale (VAS) to compare pre-treatment and post-treatment values. As all the patients in the sample was followed-up, resulting into no loss of subjects.

**Result:**

Overall, percentage reduction in VAS score at 3 months, 6 months, and 9 months was 24.45±9.09, 18.45±11.60 and 8.29±14.19%, respectively in Group I and 18.96±5.85, 7.33±8.60 and 3.20±7.39%, respectively in Group II. A statistically significant difference between two groups was observed at 3- and 6-months’ time intervals only (p<0.05). Overall, percentage reduction in WOMAC score at 3 months, 6 months and 9 months was 24.03±11.41, 17.45±9.24, and 9.49±9.80%, respectively in Group I and 11.27±5.73, 5.70±4.78, and -0.13±5.06%, respectively in Group II. At all the three-time intervals, the difference between two groups was significant statistically (p<0.001).

**Conclusion:**

This study suggested that both PRP as well as arthroscopy provide a reduction in WOMAC and VAS scores for pain among cases of knee osteoarthritis. Most effective reduction is observed at three months follow-up which thereafter tends to diminish. Of the two modalities, PRP seemed to have an edge over arthroscopic debridement, however, this efficacy was more pronounced for Kellgren-Lawrence Grade 2 as compared to Grade 3.

## Introduction

Osteoarthritis (OA) is recognised as the fourth leading reason for disability^1^. It perhaps happens to be the most common disease of joints in adults across the world^2^. Among different types of OA, Knee OA is the most common comprising nearly 80% of the OA burden^3^. As per an estimate it affects at least 19% of adults aged 45 years and older in the US^4^. Increase in life expectancy leading to increase in proportion of elderly population coupled with lifestyle changes, osteoarthritis prevalence is increasing substantially with its prevalence being doubled since the mid-20th century^5^. Osteoarthritis of the knee has great physical and economic impacts. The disease usually evolves with increasing levels of pain, mobility restriction, and physical disability^6^. Most of the patients have no option but to undergo joint replacement surgery which again is an option not without risk and limitations. Therefore, there is need for a treatment option which could avoid or delay joint replacement and make patient comfortable till surgery becomes an absolute indication. A more generic approach to current treatment methods revolves around some combination of non-pharmacological and pharmacological treatment modalities^7^. Pharmacological treatment modalities such as steroids are often associated with side effects and in the early stages of disease, physiotherapy is the preferred approach for treatment and management. On the other hand, surgical modalities such as arthroscopy involves lavage (to remove particulate material, such as cartilage fragments) and debridement (to smooth the articular surfaces). In order to evolve a successful treatment modality for knee osteoarthritis it is essential to understand the etiopathogenesis of this disease. To cope up with this degeneration it is essential that the degeneration of cartilage should either be prevented or should be compensated with adequate regeneration. That is where the role of regenerative medicine comes into picture. Regenerative medicine helps to “replace, engineer or regenerate human cells, tissues or organs in order to restore or establish the normal function”.

The platelet augmentation revolves around the concept that platelet via an intricate vesicular storage system contain critical growth factors and mediators of tissue repair pathways. As a result of tissue injury, platelet receptors are triggered through a complex interaction of cellular and non-cellular signals which in turn lead to expulsion of these growth factors from within the site of injury through the process called as degranulation. It triggers cell proliferation and subsequently leads to tissue repair response. In recent years, a preparation called Platelet rich plasma (PRP) is an emerging treatment modality classified as “Orthobiologics”. PRP is a mix of autologous blood growth factors occurring naturally and is being used in different fields of medicine owing to its ability to accelerate tissue regeneration. Alfa granules in platelets contain numerous growth factors that enhance tissue recovery dramatically by catalysing the body’s natural healing response, tissue repair processes and induce the production of new collagen by the fibroblasts, osteoblasts and chondrocytes as per need of the parent tissue. In this backdrop, we carried out this study to assess the efficacy of platelet rich plasma (PRP) as a treatment modality for osteoarthritis knee as compared to arthroscopic management.

## Materials and Methods

Clearance for carrying out the study was obtained from the Institutional Ethical Committee before starting the study. The present study was carried out as a randomised controlled clinical trial at a tertiary care centre with state-of-the-art infrastructure catering primarily to socio-economically underprivileged suburban and rural population of northern India from 2018-2020 on 70 patients. Inclusion criteria for our study were: (a) Kellgren and Lawrence grade 2 and 3, (b) Age range 40 to 60 years, (c) Failed trial of conservative treatment not more than 6 months. The exclusion criteria were: (a) Inflammatory and Crystal Arthropathies, (b) Haematological diseases, (c) Patient who had injection of hyaluronic acid or steroid in last six months, (d) Active infection, (e) Patient not willing to consent for same. Sample size is calculated on the basis of post treatment variation at 12 months in WOMAC in the two groups using the formula:


$$n=\frac{{\left(z\alpha +z\beta \right)}^{2}(\sum 1^{2}+\sum 2^{2})}{d^{2}}$$

Where Σ1 = 19.69

Σ2 =14.91 the SD of WOMAC of two group at 12 months

- Type I error α = 5%
- Type II error β = 20% for 80% power of study.

Data loss = 20%

**Fig 1:**
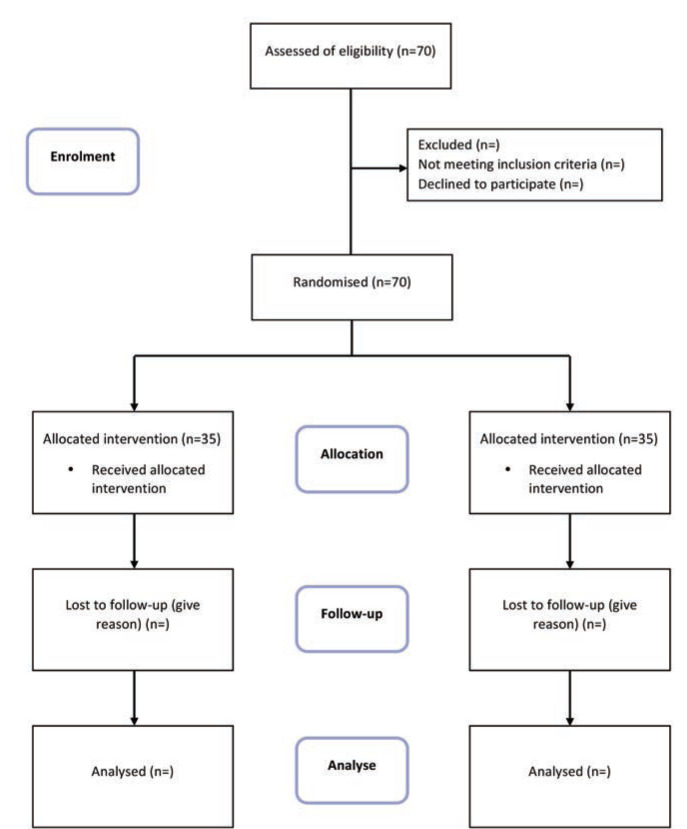
Flow chart of randomised control study

The sample size comes out to be 35 in each group. Only those providing consent to participate in the study were enrolled in the study. Patient were allocated to two groups using computer generated random numbers. Group I were administered Intra-articular injection of PRP (n=35), and Group II were subjected to arthroscopy (n=35) study is carried out as per flow chart (Fig.1).

In Group I, the PRP required for injection was prepared and provided by the Department of Pathology ELMC&H Lucknow (UP), India. About 20cc of whole blood was withdrawn under aseptic precautions atraumatically from antecubital vein, this whole blood mixed with 2.8ml of Acid Citrate Dextrose solution (ACD solution) in sterile vials, because ACD beside acting as an anticoagulant also maintains the intra platelet signal transduction mechanism during PRP preparation and thus improves overall responsiveness of platelets (Slichter and Harker, 1976)^8^.

The tubes were then centrifuged using Remi 8c centrifuge model for 15min at 1500rpm on a table top centrifuge and the blood was separated into PRP and residual red blood cells with the Buffy coat. The PRP was then extracted through a pipette and transferred to a test tube. After waiting for one hour at 20° - 22° (air-conditioned room) so that platelets come in resting phase (Makroo RN, 2014)^9^. The final product 4ml - 5ml of PRP was injected in knee by aseptic technique without prior activation by mean of pharmacological agents as PRP presents higher percentage of activated platelets. Platelets counts was done by automated machines in whole blood and in PRP. In humans, the typical baseline blood platelets count is approximately 150,000/μL - 350,000/μL while in PRP, concentration of platelets should increase 3 - 5 times than that in whole blood for proper effect. The mean platelet count in the whole blood was 241,000/μL and mean platelet count in the PRP was 1,019,000/μL. After taking informed and written consent, patient was shifted to minor O.T. Patient was placed in supine position with knee in full extension. Under aseptic condition, 4ml - 5ml of PRP was injected in knee through supralateral approach with a 22-gauge needle without local anaesthesia. Knee immobilised for 8-10min and discharged after half an hour of observation. Tablet paracetamol (650mg) was given stat in patients who experienced pain at injection site after 10min. All patients were asked to stop medications 48hrs before follow-up assessment.

In Group II, the patient was clinically examined and prepared for arthroscopy by anaesthetic staff. Suitable anaesthesia was provided after obtaining an informed consent. Patient was moved to Orthopaedic Operating theatre. A small incision called portals in the knee was made to insert the arthroscope. Several other incisions were required to see other parts of the joint or to insert other instruments. These incisions were small enough to be closed with one or two stitches, or with narrow strips of sterile adhesive tape. An arthroscopic awl was used to make micro fractures in the subchondral bone till fresh subchondral bleeding occurred. In Debridement procedure damaged portions of articular cartilage, meniscus, synovial membrane or ligaments found within the joint were excised. The joint was visualised and irrigated with normal saline or lactated ringer’s solution. Patient was shifted to recovery room for a few hours.

Patient was prescribed medication to relieve pain and inflammation. Patient was advised to take rest, compress, and elevate the joint for several days to reduce swelling and pain and if required use splints, slings or crutches. Patient was advised to immediately inform if any complications like fever, pain not helped by medication, drainage from incision site, redness or swelling, new numbness or tingling was encountered.

In both the groups, pre-operative pain was assessed using the VAS with the patient performing five active tests: (a) straight leg raising, (b) knee flexion with the patient lying supine, (c) knee extension with the patient sitting on the couch, (d) knee flexion with the patient sitting on the couch, and (e) five steps walking.

Scoring system were used to assess the post treatment condition of patient before intervention and at follow-ups at three, six and nine months. The scoring system were: (a) Visual Analog score (VAS) for Knee pain, (b) The Western Ontario and McMaster Universities Arthritis Index (WOMAC). WOMAC index is a self-administered questionnaire consisting of 24 items divided into three subscales: Pain (5 items), Stiffness (2 items), Physical Function (17 items).

The statistical analysis was done using SPSS [Statistical Package for Social Sciences] Version 21.0 statistical Analysis Software. The values were represented in Number (%) and Mean±SD.

## Results

The present study was conducted in the Department of Orthopaedics, Era’s Lucknow Medical College and Hospital to compare the clinical results of application of Arthroscopy versus Intraarticular platelet rich plasma injection in patients of osteoarthritis of knee. A total of 70 cases of osteoarthritis scheduled management fulfilling the inclusion criteria and giving consent for inclusion in the study were enrolled. These patients were randomly allocated to two equal groups by computer generated random number technique. Patients were distributed in two groups as Group 1 (n=35) and Group 2 (n=35). Following table shows comparison of general profile and disease characteristics of patients of above two groups (Table I)

**Table I:** Comparison of General Profile and Disease Characteristics of patients in two groups

		Group I (n=35)		Group I (n=35)		Statistical significance
1.	Mean age±SD (Range)	53.23±7.81 (40-60)		54.80±5.74 (44-60)		‘t’ = 0.960; p = 0.341
		No.	%	No.	%	
2.	Gender					
	Male	11	31.4	13	37.1	χ2 = 0.254; p = 0.615
	Female	24	68.6	22	62.9	
3.	Habitat					
	Rural	5	14.3	12	34.3	χ2 = 3.807; p = 0.051
	Urban	30	85.7	23	65.7	
4.	Occupation					
	Business	8	22.9	8	22.9	χ2 = 3.287; p = 0.349
	Farmer	3	8.6	2	5.7	
	Housewife	24	68.6	22	62.9	
	Shopkeeper	0	0.0	3	8.6	
5.	Side					
	Bilateral	28	80.0	24	68.6	χ2 = 1.641; p = 0.440
	Left only	3	8.6	3	8.6	
	Right only	4	11.4	8	22.9	
6.	Varus deformity	14	40.0	18	51.4	χ2 = 0.921; p = 0.337
7.	Limitation of extension	11	31.4	17	48.6	χ2 = 2.143; p = 0.143
8.	Swelling	21	60.0	18	51.4	χ2 = 0.521; p = 0.470
9.	Mean duration of symptoms±SD (Range) in months	6.66±4.35 (1-18)		6.86±4.88 (1-24)		‘t’ = 0.182; p = 0.857
10.	Mean BMI±SD (Range) in kg/m2	28.88±2.50 (22.50-33.20)		28.09±3.03 (23.50-35.10)		‘t’ = 1.197; p = 0.236
11.	Mean Pre-op. WOMAC±SD (Range)	55.97±6.24 (45-72)		58.17±6.89 (45-72)		‘t’ = 9.203; p = 0.166
12.	Mean Pre-op. VAS±SD (Range)	6.06±0.73 (5-8)		6.43±0.85 (5-8)		‘t’ = 1.966; p = 0.053
13.	Nutr. Status (Asian Criteria)	No.	%	No.	%	
	Normal (18.5-22.9)	1	2.9	0	0.0	χ2 = 3.155; p = 0.368
	Overweight (23-24.9)	2	5.7	6	17.1	
	Pre-obese (25-29.9)	18	51.4	16	45.7	
	Obese type I (30-40.0)	14	40.0	13	37.1	

At the time of enrolment, out of 70 patients 33 (47.1%) were assessed as KL Grade 2 and rest 37 (52.9%) as KL Grade 3. Though proportion of KL Grade 2 patients was higher in Group I (54.3% vs. 40.0%) but this difference was not found to be significant statistically (Table II).

**Table II:** Comparison of two groups according to Pre-intervention KL Grade

SN	Grade	Group I (n=35)		Group II (n=35)	
		No. %	No. %	No. %	No. %
1.	KL Grade 2	19	54.3	14	40.0
2.	KL Grade 3	16	45.7	21	60.0

χ2 = 1.433; p = 0.231

Pre-intervention WOMAC score of KL Grade 2, KL Grade 3 and overall patients of above two groups were found to be comparable (Table III). Pre-intervention VAS score of KL Grade 2, KL Grade 3 and overall patients of above two groups were found to be comparable (Table IV). At all the follow-up periods (three months, six months, nine months) WOMAC score of patients of Group II was found to be higher as compared to Group I (irrespective of KL Grade). Though differences in mean WOMAC score of patients of above two groups were not found to be significant for KL Grade 3 cases (Table V).

**Table III:** Comparison of two groups according to Pre-intervention WOMAC scores

SN	Variable	Group I			Group II			Statistical significance	
		n	Mean	SD	n	Mean	SD	‘t’	‘p’
1.	KL Grade 2	19	52.32	4.03	14	54.36	7.69	-0.991	0.330
2.	KL Grade 3	16	60.31	5.64	21	60.71	5.02	-0.229	0.820
3.	Overall	35	55.97	6.24	35	58.17	6.89	-1.400	0.166

**Table IV:** Comparison of two groups according to Pre-intervention VAS scores

SN	Variable	Group I			Group II			Statistical significance	
		n	Mean	SD	n	Mean	SD	‘t’	‘p’
1.	KL Grade 2	19	5.63	0.60	14	6.00	1.11	-1.230	0.228
2.	KL Grade 3	16	6.56	0.51	21	6.71	0.46	-0.944	0.352
3.	Overall	35	6.06	0.73	35	6.43	0.85	-1.966	0.053

**Table V:** Comparison of post-intervention WOMAC scores at different follow-up intervals

SN	Follow-up interval	Group I			Group II			Statistical significance	
		n	Mean	SD	n	Mean	SD	‘t’	‘p’
1.	At 3 months								
	KL Grade 2	19	35.63	2.71	14	47.43	7.52	-6.330	<0.001
	KL Grade 3	16	51.19	6.91	21	54.43	4.93	-1.667	0.104
	Overall	35	42.74	9.32	35	51.63	6.93	4.528	<0.001
2.	At 6 months								
	KL Grade 2	19	40.11	2.77	14	50.93	7.28	-5.950	<0.001
	KL Grade 3	16	53.75	6.16	21	57.43	4.99	-2.009	0.052
	Overall	35	46.34	9.32	35	54.83	6.73	4.709	<0.001
3.	At 9 months								
	KL Grade 2	19	44.32	4.22	14	53.64	7.33	-4.618	<0.001
	KL Grade 3	16	58.56	6.87	21	61.19	4.40	-1.416	0.166
	Overall	35	50.83	9.06	35	58.17	6.78	3.838	<0.001

At all the follow-up periods (three months, six months, nine months) VAS score of patients of Group II was found to be higher as compared to Group I (irrespective of KL Grade). Difference in VAS score between above two groups were not found to be significant at 3 months among KL Grade 3 cases, and at 9 months when evaluated separately for KL Grade 2 and Grade 3, but significant differences were found for overall patients at follow-up at 9 months (Table VI).

**Table VI:** Comparison of post-intervention VAS scores at different follow-up intervals

SN	Follow-up interval	Group I			Group II			Statistical significance	
		n	Mean	SD	n	Mean	SD	‘t’	‘p’
1.	At 3 months								
	KL Grade 2	19	4.00	0.58	14	4.71	0.73	-3.149	0.004
	KL Grade 3	16	5.31	0.70	21	5.52	0.51	-1.058	0.297
	Overall	35	4.60	0.91	35	5.20	0.72	-3.052	0.003
2.	At 6 months								
	KL Grade 2	19	4.53	0.61	14	5.43	0.65	-4.089	<0.001
	KL Grade 3	16	5.44	1.09	21	6.24	0.44	-3.061	0.004
	Overall	35	4.94	0.97	35	5.91	0.66	-4.907	<0.001
3.	At 9 months								
	KL Grade 2	19	5.21	0.63	14	5.64	0.74	-1.803	0.081
	KL Grade 3	16	5.94	1.34	21	6.57	0.60	-1.936	0.061
	Overall	35	5.54	1.07	35	6.20	0.80	-2.920	0.005

Overall, percentage reduction in WOMAC score at 3 months, 6 months and 9 months was 24.03±11.41, 17.45±9.24, and 9.49±9.80%, respectively in Group I and 11.27±5.73, 5.70±4.78, and -0.13±5.06%, respectively in Group II. At all the three-time intervals, the difference between two groups was statistically significant (p<0.001). Among KL2 grade patients, percentage reduction in WOMAC score at three months, six months, nine months was 31.59±6.61, 23.11±5.60, and 15.20±5.60%, respectively in Group I and 12.78±5.56, 6.24±4.80 and 1.14±5.14%, respectively in Group II. At all the three-time intervals, the difference between two groups was significant statistically (p<0.001). Among KL3 grade patients, percentage reduction in WOMAC score at 3 months, 6 months and 9 months was 15.05±9.17, 10.72±8.19, and 2.71±9.45%, respectively in Group I and 10.26±5.75, 5.34±4.85, and -0.97±4.95%, respectively in Group II. A significant difference between two groups was observed at 6 months follow-up only (p=0.017) (Table VII).

**Table VII:** Comparison of percent reduction in WOMAC scores between two groups at different follow-up intervals

SN	Follow-up interval	Group I			Group II			Statistical significance	
		n	Mean	SD	n	Mean	SD	‘t’	‘p’
1.	3 months								
	KL Grade 2	19	31.59	6.61	14	12.78	5.56	8.621	<0.001
	KL Grade 3	16	15.05	9.17	21	10.26	5.75	1.944	0.060
	Overall	35	24.03	11.41	35	11.27	5.73	5.911	<0.001
2.	6 months								
	KL Grade 2	19	23.11	5.60	14	6.24	4.80	9.069	<0.001
	KL Grade 3	16	10.72	8.19	21	5.34	4.85	2.495	0.017
	Overall	35	17.45	9.24	35	5.70	4.78	6.678	<0.001
3.	9 months								
	KL Grade 2	19	15.20	5.62	14	1.14	5.14	7.366	<0.001
	KL Grade 3	16	2.71	9.45	21	-0.97	4.95	1.534	0.134
	Overall	35	9.49	9.80	35	-0.13	5.06	5.161	<0.001

Overall, percentage reduction in VAS score at 3 months, 6 months and 9 months was 24.45±9.09, 18.45±11.60, and 8.29±14.19%, respectively in Group I and 18.96±5.85, 7.33±8.60, and 3.20±7.39%, respectively in Group II. A statistically significant difference between two groups was observed at three- and six-months’ time intervals only (p<0.05). At nine months, though percentage reduction was higher in Group I as compared to that in Group II, yet this difference was not statistically significant (p=0.064). In KL Grade 2 patients, percentage reduction in VAS score at 3 months, 6 months and 9 months was 28.87±8.24, 19.70±5.14, and 7.24±8.81%, respectively in Group I and 20.86±6.02, 8.12±10.50, and 4.76±9.65%, respectively in Group II. A statistically significant difference between two groups was observed at 3- and 6-months’ time intervals only (p<0.05). At 9 months, though percentage reduction was higher in Group I as compared to that in Group II, yet this difference was not statistically significant (p=0.448). Among KL Grade 3 patients, percentage reduction in VAS score at 3 months, 6 months and 9 months was 19.20±7.19, 16.96±17.40 and 9.52±18.99%, respectively in Group I and 17.69±5.51, 6.80±7.31, and 2.15±5.42%, respectively in Group II. A statistically significant difference between two groups was observed at 6 months’ time interval only (p=0.016). At 3 months and 9 months, though percentage reduction was higher in Group I as compared to that in Group II, yet this difference was not significant statistically (p>0.05) (Table VIII).

**Table VIII:** Comparison of overall percent reduction in VAS Score between two groups at nine months (final follow-up)

SN	Variable	Group I			Group II			Statistical significance	
		n	Mean	SD	n	Mean	SD	‘t’	‘p’
1.	3 months								
	KL Grade 2	19	28.87	8.24	14	20.86	6.02	3.078	0.004
	KL Grade 3	16	19.20	7.19	21	17.69	5.51	0.723	0.474
	Overall	35	24.45	9.09	35	18.96	5.85	3.006	0.004
2.	6 months								
	KL Grade 2	19	19.70	5.14	14	8.12	10.50	4.189	<0.001
	KL Grade 3	16	16.96	16.40	21	6.80	7.31	2.536	0.016
	Overall	35	18.45	11.60	35	7.33	8.60	4.554	<0.001
3.	9 months								
	KL Grade 2	19	7.24	8.81	14	4.76	9.65	0.768	0.448
	KL Grade 3	16	9.52	18.99	21	2.15	5.42	1.697	0.099
	Overall	35	8.29	14.19	35	3.20	7.39	1.881	0.064

## Discussion

We conducted a randomised controlled trial in which a total of 70 patients with unilateral or bilateral osteoarthritis were equally allocated to one of the two randomised groups – Group I (n=35) patients received PRP as per protocol whereas Group II (n=35) patients underwent arthroscopic debridement. There were three major considerations while planning the study – (1) whether intra-articular use of platelet-rich plasma is feasible without complications, (2) whether platelet-rich plasma provides a clinical/functional improvement in patients with osteoarthritis knee, (3) whether the treatment response obtained for PRP is better than that obtained for arthroscopic debridement.

In our study, majority of patients (52.9%) had KL grade 3. Compared to this Kon *et al*^10^ in their study reported 57.9% patients in KL grade I-III and remaining 42.1% in grade IV. Wang-Saegusa *et al*^11^ on the other hand had 36.9% grade IV cases. However, we excluded patients of grade IV as also done by some of the other authors^12-14^. Patel *et al*^15^ in their study did not use KL-grade as criteria but instead used Ahlback grade and included patients with grades 1-3 only while excluding the more severe grade 4-5 patients. In this study, we also limited our evaluation for Grade 2 and 3 patients only.

In our study, we focused on the outcome parameters in both KL Grade 2 and KL Grade 3 patients independently as well as a combined overall outcome of both the grades. A statistical matching for WOMAC scores and VAS scores was done between both the groups at the start of study, for both the grades independently as well as a common assessment for both the grades and did not show a significant difference between two groups, thus depicting that both the groups were matched statistically for various clinic-demographic parameters as well as the outcome parameters.

At all the three follow-up intervals, for overall assessment and KL Grade mean WOMAC scores were significantly lower in PRP group as compared to arthroscopy group, however, there was no significant difference between two groups when assessed for KL Grade 3. During different follow-up intervals, mean values were minimum at three months and maximum at nine months. For VAS scores too, in both the groups mean values were minimum at three months and maximum at nine months. At all the follow-up intervals, mean VAS scores were lower in PRP group as compared to arthroscopy group for both the grades independently as well as on overall combined assessment. A statistically significant difference between two groups was observed for KL Grade 2 and overall assessment at 3 months, for both KL2 and KL3 grades independently as well as for overall assessment at 6 months and for overall combined assessment only at 9 months.

On the basis of overall assessment as well as for KL Grade 2, for WOMAC score, PRP showed a definite edge over arthroscopy, however, for KL Grade 3, the results of PRP were comparable to arthroscopy. With the passage of time, both the treatments seemed to lose their effectiveness. By 9 months, the percent reduction in WOMAC scores was only 9.49±9.80% in PRP group and -0.13±5.06% in Arthroscopic debridement group. At the last follow-up, percent reduction in VAS scores was 8.29±14.19% in PRP group as compared to 3.20±7.39% in Arthroscopic debridement group. At this time interval, a significant difference between two groups was observed only for WOMAC scores.

So far, no comparative studies between PRP and arthroscopic debridement for management of knee osteoarthritis have been carried out and the present study is the first study in that direction. However, both the methods have been used extensively for management of knee osteoarthritis, either as a prospective single arm case series or a randomised trial comparing it with other popular treatment modalities.

As far as arthroscopic debridement is concerned, its efficacy has been stated to be limited to low grade osteoarthritis and long-term improvements are out of its scope^16^. In this study, we observed the percent reduction in WOMAC scores to be negative at 9 months interval whereas for VAS scores this reduction was at nominal 3.20±7.39% on overall evaluation and 4.76±9.65%, and 2.15±5.42%, respectively for KL Grade 2 and 3. In their study Gaonkar *et al*^16^ reported improvement in only 32.1% patients undergoing arthroscopic debridement at one year interval. We found a nominal reduction in pain at 9 months, thus showing an even worse response as compared to that reported by Gaonkar *et al*^16^ in their study. Although, arthroscopy has been shown to be superior as compared to conventional drug therapy^17^, this superiority is only short-term and in long-term it has been reported to be comparable to conservative treatment^18^. The findings of present study also suggested that the efficacy of this treatment did not go beyond 9 months.

However, PRP has emerged as a potential new modality with fast relief from pain and functional outcome that is sustainable up to a substantial period of time. In present study, for both WOMAC as well as VAS scores, reduction was observed up to nine months of follow-up. Gobbi *et al*^13^ in their study found PRP to be effective up to 12 months of follow-up. In present study, we found PRP to be more effective in KL Grade 2 as compared to that in Grade 3, which is similar to the observation made by Filardo *et al*^14^ who also observed improvement trends in PRP to be favourable for low grade articular degeneration (KL Grade 2). In their study too, PRP offered a significant clinical improvement up to one year of follow-up. In present study, we also observed an improvement in both WOMAC and VAS scores up to 9 months in PRP group. In another study, Say *et al*^19^ observed PRP to be better than hyaluronic acid (HA) injections at 3 and 6 months follow-up. In present study, at both these time intervals we found PRP to be better than arthroscopic debridement. In another study, Patel *et al*^15^ compared efficacy of single injection and two injections of PRP against normal saline and observed both the regimens to be effective up to six months. In present study, we found the PRP treatment to be effective up to nine months. A number of other studies have shown sustenance of improvement in functional outcomes and pain for six months or more^20^. A number of meta-analyses have also shown it to be better as compared to other alternative therapies (generally HA injections) or placebo^21-23^.

In present study, no complication or side effect was noticed in either of two groups at any point of time. These observations are similar to the observations of Spakova *et al* (2012)12, Filardo *et al* (2012)^14^ and Say *et al* (2013)^19^ who also found PRP to be safe and effective and reported of mild pain and effusion at the injection site as the only complications.

The limitations of the present study was that the outcomes were measured only for two domains – WOMAC scores and Pain. Another limitation is use of radiograph as only means of radiographic analysis due to cost constraints and MRI cartilage analysis could be used along with radiograph. Several other studies have studied the outcomes using other criteria too. Inclusion of subjective outcome in terms of patient satisfaction could also be included as assessment criteria. Moreover, the duration of follow-up could not be extended. A longer duration of follow-up would have been able to illustrate the point of time beyond which PRP loses its efficiency as seen for Arthroscopic debridement at nine months itself. In present study, we found that improvements in WOMAC scores and Pain were sustainable in PRP group as compared up to nine months of time period. In present study, we also found that PRP had higher improvement as compared to arthroscopic debridement for both KL2 as well as KL3 grades, however, owing to limitations of sample size, this difference was not significant statistically at some follow-up intervals. Hence, further studies with a larger sample size and a longer follow-up period with inclusion of other comparative treatment modalities are recommended. Nevertheless, PRP was found to be safe and efficacious treatment and could be recommended as an alternative management modality for osteoarthritis knee.

## Conclusion

At follow-up at three months, six months, nine months: (1) WOMAC score of patients administered PRP injection were lower than those subjected to arthroscopy. Though WOMAC score of patients with moderate osteoarthritis (KL Grade 3) were comparable between the above two groups, (2) VAS score of patients administered PRP injection were lower than those subjected to arthroscopy. Though VAS score of patients with moderate osteoarthritis (KL Grade 3) were comparable between the above two groups at follow-up at 3 months. In later follow-up (nine month) patients of above two groups had comparable VAS score when evaluated separately for severity of osteoarthritis (KL Grade). At final follow-up (nine months) reduction in WOMAC score was observed among cases administered PRP while increment in WOMAC score was observed among cases subjected to arthroscopy.

Of the two treatment modalities, PRP seemed to have an edge over arthroscopic debridement, however, this efficacy was more pronounced for KL Grade 2 as compared to Grade 3. PRP was found to be safe and efficacious treatment and could be recommended as an alternative management modality for osteoarthritis knee.
